# 3,3-Dieth­oxy-5-fluoro-2,3-dihydro-1*H*-indol-2-one

**DOI:** 10.1107/S1600536812001857

**Published:** 2012-01-21

**Authors:** Ahmed Bari, Abdulrahman M. Al-Obaid, Ali Syed Saeed, Seik Weng Ng

**Affiliations:** a Department of Pharmaceutical Chemistry, College of Pharmacy, King Saud University, Riyadh 11451, Saudi Arabia; b Department of Chemistry, University of Malaya, 50603 Kuala Lumpur, Malaysia; c Chemistry Department, Faculty of Science, King Abdulaziz University, PO Box 80203 Jeddah, Saudi Arabia

## Abstract

The title ketal, C_12_H_14_FNO_3_, crystallized with two independent molecules in the asymmetric unit. In each molecule the fused ring system is essentially planar [maximum deviations of 0.0169 (11) and 0.0402 (13) Å]. The mol­ecules are each hydrogen bonded across a center of inversion into a dimer; adjacent dimers are linked by another N—H⋯O hydrogen bond, forming a chain running along [100].

## Related literature

For 3,3-dimeth­oxy­indolin-2-one, see: De & Kitagawa (1991 ▶).
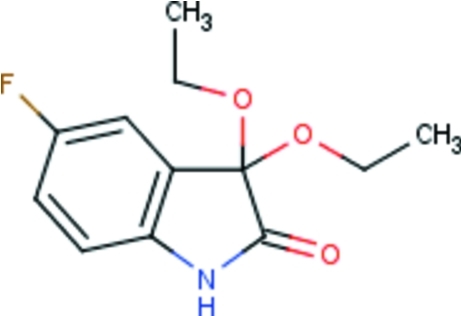


## Experimental

### 

#### Crystal data

C_12_H_14_FNO_3_
*M* *_r_* = 239.24
Triclinic, 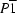

*a* = 9.3218 (6) Å
*b* = 9.4320 (5) Å
*c* = 14.1544 (8) Å
α = 100.475 (5)°
β = 104.453 (5)°
γ = 90.238 (5)°
*V* = 1183.43 (12) Å^3^
*Z* = 4
Cu *K*α radiation
μ = 0.90 mm^−1^
*T* = 100 K
0.30 × 0.20 × 0.10 mm

#### Data collection

Agilent SuperNova Dual diffractometer with an Atlas detector
Absorption correction: multi-scan (*CrysAlis PRO*; Agilent, 2011 ▶) *T*_min_ = 0.773, *T*_max_ = 0.915
8022 measured reflections
4661 independent reflections
4067 reflections with *I* > 2σ(*I*)
*R*_int_ = 0.018

#### Refinement

*R*[*F* ^2^ > 2σ(*F* ^2^)] = 0.037
*wR*(*F* ^2^) = 0.100
*S* = 1.04
4661 reflections
315 parameters
H atoms treated by a mixture of independent and constrained refinement
Δρ_max_ = 0.26 e Å^−3^
Δρ_min_ = −0.23 e Å^−3^

### 

Data collection: *CrysAlis PRO* (Agilent, 2011 ▶); cell refinement: *CrysAlis PRO*; data reduction: *CrysAlis PRO*; program(s) used to solve structure: *SHELXS97* (Sheldrick, 2008 ▶); program(s) used to refine structure: *SHELXL97* (Sheldrick, 2008 ▶); molecular graphics: *X-SEED* (Barbour, 2001 ▶); software used to prepare material for publication: *publCIF* (Westrip, 2010 ▶).

## Supplementary Material

Crystal structure: contains datablock(s) global, I. DOI: 10.1107/S1600536812001857/xu5445sup1.cif

Structure factors: contains datablock(s) I. DOI: 10.1107/S1600536812001857/xu5445Isup2.hkl

Additional supplementary materials:  crystallographic information; 3D view; checkCIF report

## Figures and Tables

**Table 1:** Hydrogen-bond geometry (Å, °)

*D*—H⋯*A*	*D*—H	H⋯*A*	*D*⋯*A*	*D*—H⋯*A*
N1—H1⋯O4	0.85 (2)	2.30 (2)	3.010 (2)	142 (2)
N1—H1⋯O1^i^	0.85 (2)	2.34 (2)	3.043 (1)	141 (2)
N2—H2⋯O1^ii^	0.87 (2)	2.28 (2)	3.002 (2)	140 (2)
N2—H2⋯O4^iii^	0.87 (2)	2.33 (2)	3.057 (2)	142 (2)

Symmetry codes: (i) 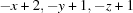; (ii) 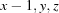; (iii) 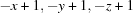.

**Fig. 1.**
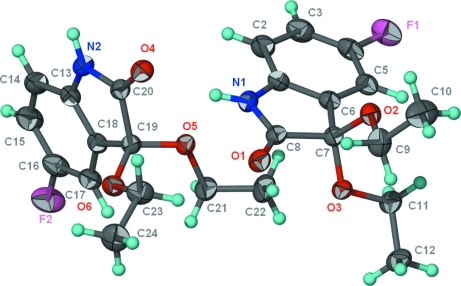
Thermal ellipsoid plot (Barbour, 2001) of the two molecules of C12H14FNO3 at the 70% probability level; hydrogen atoms are drawn as spheres of arbitrary radius.

## supplementary crystallographic information

### Comment

The parent compound, isatin, forms a ketal, 3,3-dimethoxyindolin-2-one (De & Kitagawa, 1991). The present compound, which has a fluorine atom in the ring, is expected to possess improved pharmaceutical activity. Fluorine-substituted C_12_H_14_FNO_3_ (Scheme I) exists as two independent molecules (Fig. 1) whose fused-rings are both planar. The molecules are each hydrogen-bonded across a center-of-inversion into a dimer; adjacent dimers are linked by another N–H···O hydrogen bond to form a chain running along [1 0 0] (Table 1).

### Experimental

5-Fluoroisatin (1 mol) was heated under reflux for 3 h in ethanol (20 ml) in the presence of few drops of glacial acetic acid. The solvent was removed under reduced pressure and the product was crystallized from ethanol to yield the title compound (C_12_H_14_FNO_3_) as light brown crystals.

### Refinement

Carbon-bound H-atoms were placed in calculated positions [C–H 0.95 to 0.99 Å, *U*_iso_(H) 1.2 to 1.5*U*_eq_(C)] and were included in the refinement in the riding model approximation.

The amino H-atoms were located in a difference Fourier map, and were freely refined.

The (1 - 1 1) reflection was omitted.

### Crystal data

**Table:**

C_12_H_14_FNO_3_	*Z* = 4
*M**_r_* = 239.24	*F*(000) = 504
Triclinic, *P*1	*D*_x_ = 1.343 Mg m^−^^3^
Hall symbol: -P 1	Cu *K*α radiation, λ = 1.54184 Å
*a* = 9.3218 (6) Å	Cell parameters from 3789 reflections
*b* = 9.4320 (5) Å	θ = 3.3–74.1°
*c* = 14.1544 (8) Å	µ = 0.90 mm^−^^1^
α = 100.475 (5)°	*T* = 100 K
β = 104.453 (5)°	Prism, light brown
γ = 90.238 (5)°	0.30 × 0.20 × 0.10 mm
*V* = 1183.43 (12) Å^3^	

### Data collection

**Table:**

Agilent SuperNova Dual diffractometer with an Atlas detector	4661 independent reflections
Radiation source: SuperNova (Cu) X-ray Source	4067 reflections with *I* > 2σ(*I*)
Mirror	*R*_int_ = 0.018
Detector resolution: 10.4041 pixels mm^-1^	θ_max_ = 74.3°, θ_min_ = 3.3°
ω scan	*h* = −11→8
Absorption correction: multi-scan (CrysAlis PRO; Agilent, 2011)	*k* = −11→10
*T*_min_ = 0.773, *T*_max_ = 0.915	*l* = −16→17
8022 measured reflections	

### Refinement

**Table:**

Refinement on *F*^2^	Primary atom site location: structure-invariant direct methods
Least-squares matrix: full	Secondary atom site location: difference Fourier map
*R*[*F*^2^ > 2σ(*F*^2^)] = 0.037	Hydrogen site location: inferred from neighbouring sites
*wR*(*F*^2^) = 0.100	H atoms treated by a mixture of independent and constrained refinement
*S* = 1.04	*w* = 1/[σ^2^(*F*_o_^2^) + (0.0505*P*)^2^ + 0.3265*P*] where *P* = (*F*_o_^2^ + 2*F*_c_^2^)/3
4661 reflections	(Δ/σ)_max_ = 0.001
315 parameters	Δρ_max_ = 0.26 e Å^−^^3^
0 restraints	Δρ_min_ = −0.23 e Å^−^^3^

### Fractional atomic coordinates and isotropic or equivalent isotropic displacement parameters (Å^2^)

**Table:**

	*x*	*y*	*z*	*U*_iso_*/*U*_eq_	
F1	0.65631 (10)	0.87322 (9)	0.07446 (6)	0.0377 (2)	
F2	0.13605 (10)	−0.08585 (10)	0.06203 (8)	0.0442 (2)	
O1	1.11315 (10)	0.49565 (10)	0.42494 (7)	0.0253 (2)	
O2	1.15077 (10)	0.75706 (10)	0.32386 (7)	0.0233 (2)	
O3	1.10671 (10)	0.52344 (9)	0.22746 (6)	0.0221 (2)	
O4	0.61151 (10)	0.46293 (10)	0.42320 (7)	0.0253 (2)	
O5	0.60229 (10)	0.32961 (9)	0.22563 (6)	0.0223 (2)	
O6	0.64294 (10)	0.14599 (10)	0.31984 (7)	0.0244 (2)	
N1	0.87973 (12)	0.57806 (12)	0.37342 (8)	0.0241 (2)	
H1	0.838 (2)	0.544 (2)	0.4122 (14)	0.040 (5)*	
N2	0.37762 (13)	0.35119 (12)	0.37406 (9)	0.0253 (2)	
H2	0.3385 (19)	0.410 (2)	0.4141 (14)	0.037 (5)*	
C1	0.80501 (14)	0.65008 (13)	0.29789 (10)	0.0234 (3)	
C2	0.65810 (15)	0.68508 (15)	0.27701 (12)	0.0307 (3)	
H2A	0.5932	0.6589	0.3139	0.037*	
C3	0.60863 (16)	0.76044 (16)	0.19961 (12)	0.0336 (3)	
H3	0.5084	0.7864	0.1825	0.040*	
C4	0.70630 (16)	0.79666 (15)	0.14840 (10)	0.0290 (3)	
C5	0.85398 (15)	0.76166 (14)	0.16810 (9)	0.0243 (3)	
H5	0.9187	0.7882	0.1312	0.029*	
C6	0.90179 (14)	0.68592 (13)	0.24451 (9)	0.0211 (3)	
C7	1.05296 (14)	0.63435 (13)	0.28796 (9)	0.0200 (3)	
C8	1.02300 (14)	0.56035 (13)	0.37198 (9)	0.0210 (3)	
C9	1.30237 (15)	0.73120 (17)	0.37099 (11)	0.0310 (3)	
H9A	1.3449	0.6631	0.3246	0.037*	
H9B	1.3064	0.6893	0.4308	0.037*	
C10	1.38741 (17)	0.87367 (18)	0.39909 (14)	0.0429 (4)	
H10A	1.4911	0.8606	0.4316	0.064*	
H10B	1.3441	0.9401	0.4449	0.064*	
H10C	1.3828	0.9138	0.3393	0.064*	
C11	1.13018 (16)	0.55704 (15)	0.13718 (10)	0.0278 (3)	
H11A	1.1812	0.6536	0.1504	0.033*	
H11B	1.0341	0.5572	0.0877	0.033*	
C12	1.22457 (18)	0.44256 (17)	0.09873 (11)	0.0343 (3)	
H12A	1.2436	0.4623	0.0372	0.051*	
H12B	1.1725	0.3477	0.0854	0.051*	
H12C	1.3190	0.4432	0.1485	0.051*	
C13	0.29953 (15)	0.24155 (14)	0.29679 (10)	0.0251 (3)	
C14	0.15128 (16)	0.19850 (16)	0.27473 (12)	0.0335 (3)	
H14	0.0885	0.2433	0.3136	0.040*	
C15	0.09679 (16)	0.08786 (16)	0.19405 (13)	0.0376 (4)	
H15	−0.0049	0.0563	0.1762	0.045*	
C16	0.19158 (16)	0.02415 (15)	0.14008 (12)	0.0329 (3)	
C17	0.34117 (15)	0.06529 (14)	0.16144 (11)	0.0269 (3)	
H17	0.4041	0.0188	0.1233	0.032*	
C18	0.39400 (14)	0.17753 (13)	0.24116 (10)	0.0227 (3)	
C19	0.54709 (14)	0.25080 (13)	0.28508 (9)	0.0208 (3)	
C20	0.52002 (14)	0.36945 (14)	0.37073 (9)	0.0215 (3)	
C21	0.62215 (17)	0.24781 (15)	0.13397 (10)	0.0303 (3)	
H21A	0.5248	0.2208	0.0857	0.036*	
H21B	0.6731	0.1584	0.1459	0.036*	
C22	0.7143 (2)	0.34170 (18)	0.09427 (13)	0.0470 (5)	
H22A	0.7305	0.2890	0.0318	0.071*	
H22B	0.8101	0.3680	0.1427	0.071*	
H22C	0.6624	0.4294	0.0824	0.071*	
C23	0.79515 (15)	0.19686 (16)	0.36725 (11)	0.0313 (3)	
H23A	0.7992	0.2752	0.4250	0.038*	
H23B	0.8400	0.2349	0.3199	0.038*	
C24	0.87762 (17)	0.07179 (18)	0.40046 (12)	0.0370 (3)	
H24A	0.9812	0.1032	0.4336	0.055*	
H24B	0.8740	−0.0045	0.3427	0.055*	
H24C	0.8318	0.0346	0.4469	0.055*	

### Atomic displacement parameters (Å^2^)

**Table:**

	*U*^11^	*U*^22^	*U*^33^	*U*^12^	*U*^13^	*U*^23^
F1	0.0425 (5)	0.0345 (5)	0.0326 (5)	0.0107 (4)	−0.0018 (4)	0.0134 (4)
F2	0.0342 (5)	0.0300 (5)	0.0570 (6)	−0.0055 (4)	0.0041 (4)	−0.0101 (4)
O1	0.0267 (5)	0.0309 (5)	0.0204 (4)	0.0060 (4)	0.0067 (4)	0.0089 (4)
O2	0.0220 (5)	0.0228 (4)	0.0236 (4)	−0.0025 (3)	0.0037 (4)	0.0036 (4)
O3	0.0279 (5)	0.0230 (4)	0.0174 (4)	0.0047 (4)	0.0085 (3)	0.0049 (3)
O4	0.0269 (5)	0.0275 (5)	0.0203 (4)	0.0006 (4)	0.0056 (4)	0.0019 (4)
O5	0.0273 (5)	0.0219 (4)	0.0191 (4)	0.0002 (3)	0.0088 (3)	0.0033 (3)
O6	0.0222 (5)	0.0231 (5)	0.0284 (5)	0.0053 (4)	0.0053 (4)	0.0076 (4)
N1	0.0245 (6)	0.0258 (6)	0.0255 (6)	0.0037 (4)	0.0105 (5)	0.0084 (4)
N2	0.0263 (6)	0.0246 (6)	0.0273 (6)	0.0024 (4)	0.0121 (5)	0.0035 (5)
C1	0.0248 (6)	0.0194 (6)	0.0256 (6)	0.0021 (5)	0.0058 (5)	0.0040 (5)
C2	0.0252 (7)	0.0271 (7)	0.0417 (8)	0.0041 (5)	0.0104 (6)	0.0089 (6)
C3	0.0258 (7)	0.0292 (7)	0.0430 (8)	0.0060 (6)	0.0028 (6)	0.0076 (6)
C4	0.0342 (7)	0.0222 (6)	0.0264 (7)	0.0051 (5)	−0.0012 (5)	0.0064 (5)
C5	0.0296 (7)	0.0203 (6)	0.0211 (6)	0.0006 (5)	0.0038 (5)	0.0030 (5)
C6	0.0227 (6)	0.0184 (6)	0.0199 (6)	0.0008 (5)	0.0030 (5)	0.0012 (5)
C7	0.0222 (6)	0.0203 (6)	0.0168 (6)	0.0003 (5)	0.0048 (5)	0.0022 (5)
C8	0.0242 (6)	0.0208 (6)	0.0177 (6)	0.0007 (5)	0.0062 (5)	0.0013 (5)
C9	0.0219 (7)	0.0384 (8)	0.0309 (7)	−0.0031 (6)	0.0012 (5)	0.0097 (6)
C10	0.0292 (8)	0.0405 (9)	0.0497 (10)	−0.0086 (7)	0.0063 (7)	−0.0099 (7)
C11	0.0370 (7)	0.0313 (7)	0.0189 (6)	0.0080 (6)	0.0109 (5)	0.0091 (5)
C12	0.0458 (9)	0.0385 (8)	0.0258 (7)	0.0144 (7)	0.0183 (6)	0.0112 (6)
C13	0.0264 (7)	0.0205 (6)	0.0307 (7)	0.0026 (5)	0.0096 (5)	0.0075 (5)
C14	0.0270 (7)	0.0260 (7)	0.0507 (9)	0.0022 (5)	0.0166 (6)	0.0062 (6)
C15	0.0245 (7)	0.0268 (7)	0.0599 (10)	−0.0023 (6)	0.0109 (7)	0.0037 (7)
C16	0.0303 (7)	0.0208 (6)	0.0422 (8)	−0.0020 (5)	0.0036 (6)	0.0002 (6)
C17	0.0272 (7)	0.0213 (6)	0.0319 (7)	0.0032 (5)	0.0070 (5)	0.0053 (5)
C18	0.0229 (6)	0.0199 (6)	0.0269 (6)	0.0031 (5)	0.0066 (5)	0.0079 (5)
C19	0.0225 (6)	0.0210 (6)	0.0198 (6)	0.0034 (5)	0.0060 (5)	0.0058 (5)
C20	0.0241 (6)	0.0236 (6)	0.0189 (6)	0.0049 (5)	0.0065 (5)	0.0076 (5)
C21	0.0411 (8)	0.0278 (7)	0.0224 (6)	−0.0042 (6)	0.0136 (6)	−0.0016 (5)
C22	0.0753 (12)	0.0366 (9)	0.0373 (9)	−0.0098 (8)	0.0369 (9)	−0.0035 (7)
C23	0.0218 (7)	0.0320 (7)	0.0373 (8)	0.0046 (5)	0.0028 (6)	0.0053 (6)
C24	0.0309 (8)	0.0417 (9)	0.0378 (8)	0.0095 (6)	0.0021 (6)	0.0152 (7)

### Geometric parameters (Å, °)

**Table:**

F1—C4	1.3667 (16)	C10—H10A	0.9800
F2—C16	1.3631 (16)	C10—H10B	0.9800
O1—C8	1.2233 (16)	C10—H10C	0.9800
O2—C7	1.4054 (15)	C11—C12	1.5065 (19)
O2—C9	1.4467 (16)	C11—H11A	0.9900
O3—C7	1.4041 (14)	C11—H11B	0.9900
O3—C11	1.4378 (15)	C12—H12A	0.9800
O4—C20	1.2231 (16)	C12—H12B	0.9800
O5—C19	1.4021 (15)	C12—H12C	0.9800
O5—C21	1.4387 (15)	C13—C14	1.3806 (19)
O6—C19	1.4080 (15)	C13—C18	1.3920 (18)
O6—C23	1.4477 (16)	C14—C15	1.387 (2)
N1—C8	1.3514 (17)	C14—H14	0.9500
N1—C1	1.4093 (17)	C15—C16	1.378 (2)
N1—H1	0.850 (19)	C15—H15	0.9500
N2—C20	1.3513 (17)	C16—C17	1.389 (2)
N2—C13	1.4074 (18)	C17—C18	1.3858 (19)
N2—H2	0.868 (19)	C17—H17	0.9500
C1—C2	1.3816 (19)	C18—C19	1.5143 (17)
C1—C6	1.3892 (18)	C19—C20	1.5645 (17)
C2—C3	1.397 (2)	C21—C22	1.501 (2)
C2—H2A	0.9500	C21—H21A	0.9900
C3—C4	1.374 (2)	C21—H21B	0.9900
C3—H3	0.9500	C22—H22A	0.9800
C4—C5	1.388 (2)	C22—H22B	0.9800
C5—C6	1.3862 (18)	C22—H22C	0.9800
C5—H5	0.9500	C23—C24	1.494 (2)
C6—C7	1.5109 (17)	C23—H23A	0.9900
C7—C8	1.5638 (17)	C23—H23B	0.9900
C9—C10	1.494 (2)	C24—H24A	0.9800
C9—H9A	0.9900	C24—H24B	0.9800
C9—H9B	0.9900	C24—H24C	0.9800
			
C7—O2—C9	115.97 (10)	C11—C12—H12B	109.5
C7—O3—C11	116.02 (10)	H12A—C12—H12B	109.5
C19—O5—C21	115.78 (10)	C11—C12—H12C	109.5
C19—O6—C23	115.62 (10)	H12A—C12—H12C	109.5
C8—N1—C1	111.67 (11)	H12B—C12—H12C	109.5
C8—N1—H1	124.2 (12)	C14—C13—C18	122.18 (13)
C1—N1—H1	124.0 (12)	C14—C13—N2	127.65 (13)
C20—N2—C13	111.84 (11)	C18—C13—N2	110.17 (11)
C20—N2—H2	122.1 (12)	C13—C14—C15	117.98 (14)
C13—N2—H2	125.4 (12)	C13—C14—H14	121.0
C2—C1—C6	122.39 (13)	C15—C14—H14	121.0
C2—C1—N1	127.41 (12)	C16—C15—C14	119.40 (13)
C6—C1—N1	110.20 (11)	C16—C15—H15	120.3
C1—C2—C3	117.46 (13)	C14—C15—H15	120.3
C1—C2—H2A	121.3	F2—C16—C15	118.37 (13)
C3—C2—H2A	121.3	F2—C16—C17	118.15 (13)
C4—C3—C2	119.40 (13)	C15—C16—C17	123.48 (14)
C4—C3—H3	120.3	C18—C17—C16	116.68 (13)
C2—C3—H3	120.3	C18—C17—H17	121.7
F1—C4—C3	118.38 (13)	C16—C17—H17	121.7
F1—C4—C5	117.76 (13)	C17—C18—C13	120.27 (12)
C3—C4—C5	123.85 (13)	C17—C18—C19	131.42 (12)
C6—C5—C4	116.34 (13)	C13—C18—C19	108.30 (11)
C6—C5—H5	121.8	O5—C19—O6	113.37 (10)
C4—C5—H5	121.8	O5—C19—C18	116.90 (10)
C5—C6—C1	120.55 (12)	O6—C19—C18	107.38 (10)
C5—C6—C7	130.95 (12)	O5—C19—C20	103.75 (9)
C1—C6—C7	108.46 (11)	O6—C19—C20	113.04 (10)
O3—C7—O2	113.26 (10)	C18—C19—C20	101.92 (10)
O3—C7—C6	116.93 (10)	O4—C20—N2	126.88 (12)
O2—C7—C6	107.15 (10)	O4—C20—C19	125.53 (11)
O3—C7—C8	103.67 (9)	N2—C20—C19	107.57 (11)
O2—C7—C8	113.47 (10)	O5—C21—C22	107.26 (11)
C6—C7—C8	101.94 (10)	O5—C21—H21A	110.3
O1—C8—N1	126.69 (12)	C22—C21—H21A	110.3
O1—C8—C7	125.62 (11)	O5—C21—H21B	110.3
N1—C8—C7	107.67 (10)	C22—C21—H21B	110.3
O2—C9—C10	107.04 (12)	H21A—C21—H21B	108.5
O2—C9—H9A	110.3	C21—C22—H22A	109.5
C10—C9—H9A	110.3	C21—C22—H22B	109.5
O2—C9—H9B	110.3	H22A—C22—H22B	109.5
C10—C9—H9B	110.3	C21—C22—H22C	109.5
H9A—C9—H9B	108.6	H22A—C22—H22C	109.5
C9—C10—H10A	109.5	H22B—C22—H22C	109.5
C9—C10—H10B	109.5	O6—C23—C24	107.58 (12)
H10A—C10—H10B	109.5	O6—C23—H23A	110.2
C9—C10—H10C	109.5	C24—C23—H23A	110.2
H10A—C10—H10C	109.5	O6—C23—H23B	110.2
H10B—C10—H10C	109.5	C24—C23—H23B	110.2
O3—C11—C12	107.09 (11)	H23A—C23—H23B	108.5
O3—C11—H11A	110.3	C23—C24—H24A	109.5
C12—C11—H11A	110.3	C23—C24—H24B	109.5
O3—C11—H11B	110.3	H24A—C24—H24B	109.5
C12—C11—H11B	110.3	C23—C24—H24C	109.5
H11A—C11—H11B	108.6	H24A—C24—H24C	109.5
C11—C12—H12A	109.5	H24B—C24—H24C	109.5
			
C8—N1—C1—C2	179.41 (13)	C20—N2—C13—C14	−176.79 (14)
C8—N1—C1—C6	−1.50 (15)	C20—N2—C13—C18	2.93 (15)
C6—C1—C2—C3	−0.6 (2)	C18—C13—C14—C15	−0.2 (2)
N1—C1—C2—C3	178.38 (13)	N2—C13—C14—C15	179.54 (14)
C1—C2—C3—C4	−0.2 (2)	C13—C14—C15—C16	0.8 (2)
C2—C3—C4—F1	−178.54 (13)	C14—C15—C16—F2	179.01 (14)
C2—C3—C4—C5	0.6 (2)	C14—C15—C16—C17	−0.5 (2)
F1—C4—C5—C6	179.04 (11)	F2—C16—C17—C18	179.93 (12)
C3—C4—C5—C6	−0.1 (2)	C15—C16—C17—C18	−0.6 (2)
C4—C5—C6—C1	−0.75 (18)	C16—C17—C18—C13	1.27 (19)
C4—C5—C6—C7	−177.98 (12)	C16—C17—C18—C19	−179.51 (13)
C2—C1—C6—C5	1.1 (2)	C14—C13—C18—C17	−0.9 (2)
N1—C1—C6—C5	−178.01 (11)	N2—C13—C18—C17	179.32 (12)
C2—C1—C6—C7	178.93 (12)	C14—C13—C18—C19	179.68 (13)
N1—C1—C6—C7	−0.22 (14)	N2—C13—C18—C19	−0.06 (14)
C11—O3—C7—O2	−62.62 (14)	C21—O5—C19—O6	63.57 (14)
C11—O3—C7—C6	62.73 (14)	C21—O5—C19—C18	−62.18 (15)
C11—O3—C7—C8	173.97 (10)	C21—O5—C19—C20	−173.44 (10)
C9—O2—C7—O3	−50.93 (14)	C23—O6—C19—O5	50.91 (14)
C9—O2—C7—C6	178.63 (10)	C23—O6—C19—C18	−178.41 (11)
C9—O2—C7—C8	66.91 (14)	C23—O6—C19—C20	−66.80 (14)
C5—C6—C7—O3	−68.73 (17)	C17—C18—C19—O5	66.09 (18)
C1—C6—C7—O3	113.78 (12)	C13—C18—C19—O5	−114.61 (12)
C5—C6—C7—O2	59.62 (17)	C17—C18—C19—O6	−62.58 (17)
C1—C6—C7—O2	−117.87 (11)	C13—C18—C19—O6	116.72 (11)
C5—C6—C7—C8	179.04 (13)	C17—C18—C19—C20	178.39 (13)
C1—C6—C7—C8	1.55 (13)	C13—C18—C19—C20	−2.31 (13)
C1—N1—C8—O1	−176.06 (12)	C13—N2—C20—O4	174.25 (12)
C1—N1—C8—C7	2.48 (14)	C13—N2—C20—C19	−4.36 (14)
O3—C7—C8—O1	54.27 (15)	O5—C19—C20—O4	−52.78 (15)
O2—C7—C8—O1	−69.00 (16)	O6—C19—C20—O4	70.43 (15)
C6—C7—C8—O1	176.13 (12)	C18—C19—C20—O4	−174.63 (12)
O3—C7—C8—N1	−124.28 (11)	O5—C19—C20—N2	125.86 (11)
O2—C7—C8—N1	112.44 (12)	O6—C19—C20—N2	−110.93 (12)
C6—C7—C8—N1	−2.42 (13)	C18—C19—C20—N2	4.01 (12)
C7—O2—C9—C10	177.52 (12)	C19—O5—C21—C22	−166.65 (13)
C7—O3—C11—C12	164.91 (11)	C19—O6—C23—C24	178.89 (11)

### Hydrogen-bond geometry (Å, °)

**Table:**

*D*—H···*A*	*D*—H	H···*A*	*D*···*A*	*D*—H···*A*
N1—H1···O4	0.85 (2)	2.30 (2)	3.010 (2)	142 (2)
N1—H1···O1^i^	0.85 (2)	2.34 (2)	3.043 (1)	141 (2)
N2—H2···O1^ii^	0.87 (2)	2.28 (2)	3.002 (2)	140 (2)
N2—H2···O4^iii^	0.87 (2)	2.33 (2)	3.057 (2)	142 (2)

Symmetry codes: (i) −*x*+2, −*y*+1, −*z*+1; (ii) *x*−1, *y*, *z*; (iii) −*x*+1, −*y*+1, −*z*+1.
